# The comparative study of chronically ill and healthy children and adolescents in the light of their general mental health

**DOI:** 10.1038/s41598-024-57442-y

**Published:** 2024-03-21

**Authors:** Péter Boris, Karolina Eszter Kovács, Beáta Erika Nagy

**Affiliations:** 1 Laki Kálmán Doctoral School, University of Debrecen, Debrecen, 4032 Hungary; 2 Faculty of Arts, Institute of Psychology, University of Debrecen, Debrecen, 4032 Hungary; 3 Pediatric Rehabilitation, Pediatric Psychology and Psychosomatic Unit, Faculty of Medicine, Institute of Pediatrics, University of Debrecen, Debrecen, 4032 Hungary

**Keywords:** Chronic disease, Child and adolescent compliance, Attitude assessment, Doctor-patient relationship, Psychology, Health care

## Abstract

Children's hospitalisation is difficult for the family and the immediate environment. In these cases, the provision of psychological support is particularly important. Chronically ill children who are regularly hospitalised are in a particularly difficult situation, often feeling vulnerable. Our research aims to explore and analyse in detail the psychological state, attitudes and mental health of chronically ill children and to compare patient groups (children receiving care in pulmonology, gastroenterology, onco-haematology and rehabilitation) to understand the interacting factors, which may be of great importance for quality patient care and for measures to improve patient care in the future. We studied chronically ill children (N = 107) aged 10 to 18 years (M = 14.3; SD = 2.0), cared for by the Department of Paediatric Rehabilitation, Paediatric Psychiatry and Psychosomatics of the University of Debrecen Clinical Centre, the second largest paediatric institution in Hungary. In our survey, sociodemographic questions, the Connor–Davidson Resilience Scale, the Satisfaction With Life Scale, the Cantril Ladder, the Non-Productive Thoughts Questionnaire, the Problematic Internet Use Questionnaire, the Drawing version of Pictorial Representation of Illness Self-Measure (PRISM-D), the Beck Depression Inventory—Shortened Scale, the Illness Intrusiveness Ratings Scale, the Spielberger State-Trait Anxiety Questionnaire—Child Version and the Strength and Difficulty Questionnaire were applied. One-way analysis of variance (ANOVA) was used to examine differences between groups, and Pearson rank correlation analysis was used to measure the relationships between individual variables. The results show significant differences between patient groups in terms of resilience, depression, nonproductive thoughts, problematic internet use, anxiety and coping, but no consistent pattern in the development of scores. In addition, for some psychological correlates, the role of sociodemographic background also showed significant results. The practical utility of our study is that using questionnaire methods to map patient satisfaction, compliance, and patient attitudes will provide regarding the factors that influence the mental health status of children living with chronic illnesses. In the light of this, additional methods and tools can be included to improve the quality of healthcare and to develop a set of procedures that will serve the intended purpose.

## Introduction

Chronic diseases permanently change the way we live and require constant adaptation. Chronicity is characterised by an unpredictable course of illness, reduced physical performance/ability, changes in physical appearance, persistent dependence on specialists, treatment, technical aids and physical assistance, and changes in life prospects^1^, and therefore the impact of chronic illness on personality and psychological well-being is significant, providing a good basis for the relevance of research in this area.

Chronic disease management requires a long-term care plan, and treatment adherence is paramount to achieving better health outcomes, quality of life and cost-effective health care^2^. The World Health Organization's guideline on adherence behaviour states that "increasing adherence may have a greater impact on health than improving specific medical therapy"^3^. However, chronic disease and its treatments place broad behavioural demands on adolescents. Precisely scheduled daily medication, consumption of special dietary products, regular exercise, regular visits to health care providers and monitoring blood glucose levels are just some of the demands. Asthma attacks and other attacks, periods of intense pain, immobility and unpredictable situations can aggravate their condition. These needs affect almost every aspect of adolescents' lives, including school, eating, sports, work and travel. Self-care problems are, therefore, common among children and adolescents with chronic illnesses. Non-cooperation with the doctor and non-adherence to recommendations and instructions is a major problem. Previous studies have shown that about 50% of chronically ill adolescents do not adhere to care recommendations, but this has a significant impact on health^4^. In the United States, it is estimated to cause about 125,000 deaths, at least 10% of hospitalisations, and a significant increase in morbidity and mortality^2^.

Recent literature on medical communication and disease behaviour has consistently distinguished between disease process and disease representation, whereby disease process refers to the phenomena that can be detected by physical examination with instruments, and disease state refers to how the patient represents the disease in his or her cognition. The clinician and the treatment team must gather sufficient information about both to ensure the effectiveness of treatment^5^, which can also provide an adequate physical and psychological basis for self-management of the disease. The importance of understanding and promoting self-management in the care of chronic diseases in children is becoming increasingly evident, not only through research and clinical efforts but also through the introduction of new health policies. However, this is not so simple, as self-management is a complex phenomenon determined by the influence of several factors, both separately and in combination. The theory of Modi et al.^6^ provides an excellent illustration of self-management. Self-management comprises three interdependent parts: self-management behaviours, contextual variables in four domains (individual, family, health system, community influences) that affect implementing these behaviours, and processes that link influences to self-management behaviours. Thus, compliance, adherence and self-management are not measured and examined in isolation but in context.

Our study aims to map children's and adolescents' sociodemographic characteristics, their reactive behaviour, subjective somatic health complaints, health status, illness representation, life satisfaction, vision, presence of non-productive thoughts, problematic internet use and illness burden. Our research started in 2021 at the Department of Paediatric Rehabilitation, Psychology and Psychosomatics, University of Debrecen, University of Debrecen. During the research, we set two research questions:

1. Is there a difference in the development of mental health indicators in the patient groups?
2. Can any differences in the mental health-related variables alongside the sociodemographic background variables?
3. Is there a correlation between the different mental health indicators?

Based on the research questions, we formulated the following hypotheses:

H1: Significant differences can be experienced regarding the mental health status (resilience, coping, satisfaction with life, future, anxiety, non-productive thoughts, problematic internet use, depression, and illness representation) between the healthy and chronically ill children; we hypothesise that children living with chronic illnesses report worse mental health status.

H2: Significant differences can be found in the mental health status of children regarding the sociodemographic background variables; girls, children whose parents have lower educational attainment and those who received their diagnosis in a later life stage can be experienced with worse mental health status.

H3: We hypothesise that positive mental health-related variables (resilience, coping, satisfaction with life, future) are in positive relationship with the other positive mental health-related variables and are in negative connection with negative variables (anxiety, non-productive thoughts, problematic internet use, depression, and illness representation).

### Psychological characteristics of children with chronic illness

Children with chronic illnesses frequently manifest non-productive thoughts, reflecting the intricate interplay between their health challenges and cognitive processes^7^. These non-productive thoughts often encompass heightened health-related anxiety, fear of stigmatization, and concerns about missed academic or social opportunities^8^. Research underscores the impact of these cognitive patterns on mental health outcomes and overall well-being. Interventions targeting cognitive restructuring and fostering adaptive coping mechanisms become pivotal in mitigating the detrimental effects of non-productive thoughts^9^.

The prevalence of anxiety and depressive symptoms among children with chronic illnesses is a well-documented concern in the realm of pediatric healthcare. Extensive research consistently reveals heightened rates of depression in this population, attributable to the prolonged nature of their health challenges and associated psychosocial stressors^10,11^. Elevated symptomatology often compromises the quality of life and adherence to medical regimens, necessitating targeted interventions^12,13^. Recognition of the widespread nature of depressive symptoms in children with chronic illnesses underscores the urgency for integrated mental health support within healthcare frameworks^14^. A comprehensive approach is indispensable, aiming to alleviate the burden of depression and enhance the overall well-being of these vulnerable individuals.

The relevance of addictions, particularly problematic internet use, among children with chronic illnesses is a critical facet requiring scholarly attention. These vulnerable individuals may turn to excessive online engagement as a coping mechanism, potentially exacerbating mental health challenges^15^. Addressing this intersection demands a nuanced understanding of the intricate relationship between chronic illness and addictive behaviours. The issue of problematic internet use encompasses individuals who excessively devote their time to online activities, disregarding their other daily responsibilities. This behavior ultimately leads to negative effects on both their physical and psychological health. Fluctuations in academic performance, disruptions in sleep and nutrition, social isolation, and involvement in risky behaviors linked to substance abuse are all correlated with problematic internet use^16,17^.

Resilience and coping holds profound relevance among children grappling with chronic illnesses, serving as a crucial determinant of their adaptive capacity and overall well-being^18^. Extensive empirical evidence highlights resilience as a multifaceted construct, encompassing psychological, social, and cognitive dimensions^19^. Understanding and fostering resilience in this population prove essential, as it mitigates the adverse psychological impact of chronic illnesses, facilitates effective coping strategies, and enhances overall quality of life^20,21^. Moreover, resilient children exhibit a greater capacity to navigate healthcare complexities, adhere to treatment regimens, and sustain positive social relationships.

### The role of sociodemographic background variables in the development of mental health indicators

The impact of sociodemographic background on the psychological characteristics of children grappling with chronic illnesses is a multifaceted domain warranting comprehensive examination. Socioeconomic status significantly influences access to healthcare resources, shaping the child's psychological resilience and coping strategies^22^. Economic disparities may contribute to heightened stress, impacting mental well-being^23^. Cultural influences play a pivotal role in shaping the perception of illness, attitudes toward treatment, and the utilization of support networks^24^. Family dynamics, influenced by sociodemographic factors, contribute to the child's psychosocial environment, affecting emotional regulation and adaptive coping^25^. Moreover, geographic location and community resources further shape the psychological landscape of these children. Rural and urban disparities may result in differential access to specialized healthcare services and support systems^26^. Educational opportunities and social integration, influenced by sociodemographic factors, are integral components impacting psychological development.

The sociodemographic background of children facing chronic illnesses holds profound relevance in understanding variations in resilience and coping mechanisms. Factors such as socioeconomic status, cultural influences, and familial support significantly impact how children navigate persistent health challenges^27^. Socioeconomic disparities may shape access to resources and support networks, influencing resilience levels^28^. Recognizing the nuanced interplay between sociodemographic factors, resilience, and coping is imperative for healthcare providers, educators, and policymakers. Tailored interventions must consider these variables, ensuring equitable support for children across diverse sociodemographic backgrounds as they confront the intricate complexities of chronic illnesses.

## Methods

### Sample

Our study included children aged 10–18 years with a chronic disease who regularly attend the Paediatric Clinic's gastroenterology, pulmonology, onco-haematology and paediatric rehabilitation departments and one of their parents/guardians. The questionnaires were administered in the personal presence of the PhD student conducting the research, each time under the supervision of the clinical psychologist professor conducting the research. Respondents were asked to complete the questionnaire on a voluntary basis. In all cases, intellectual disability and illiteracy were excluded from the study. The assumption that a 10-year-old child is able to complete a test package of this structure and length independently was also an important factor in the selection of the age group.

During the research, convenience sampling method was used to gather participants for a questionnaire survey, where they completed several questionnaires. The inclusion criteria of the research group were the presence of any chronic disorder, being under medical treatment in the Paediatric Clinic's of the University of Debrecen Clinical Centre and being less than 18 years old. The control group consisted of the peers of such children (below 18 years) not having any chronic disorder and not being under medical treatment. Having any kind of chronical illness was considered as exclusion criteria regarding the control group and being older than 18 years for both groups.

The total research sample consists of children and caregivers (294 children and one parent per child), of whom 107 chronically ill children and parents had their questionnaires analysed while they were complete. Among the parents and children, some decided not to continue participating in the study during the test administration: 53 cases occurred, the reasons being difficulties in completing the test, tiredness, train departure, etc. It is also worth mentioning that the pandemic period of COVID-19 caused by the SARS-CoV-2 virus, a pandemic infectious disease, also made it difficult to take the test on several occasions when people preferred to spend the minimum time in and around the clinics. Once they had undergone the necessary medical examination, they tried to leave as soon as possible. Overall, the total number of children considered for this current research was N = 207 (see further details in Table 1).

**Table 1:** Sociodemographic characteristics of the study sample.

	Main	%
No		
Boy	99	47.8
Girl	108	52.2
Residence		
Capital	2	1.0
County Seat	45	21.7
Big City	16	7.7
Small Town	123	59.4
Village	21	10.1
Mother's Education		
Primary	15	7.8
Secondary	81	42.2
Tertiary	96	50.0
Father's Education		
Primary	18	9.5
Secondary	97	51.1
Tertiary	75	39.5
Financial Situation		
Low	5	84.5
Medium	174	12.1
High	25	12.1
Patient Group		
Healthy	100	48.3
Gastroenterology Patients	34	16.4
Onco-Haematology Patients	21	10.1
Pulmonology Patients	19	9.3
Patients Requiring Rehabilitation	33	15.9

### Instruments

With children and adolescents, we took a pre-assembled set of tests to assess the components of the personality profile of children with chronic illnesses that might be related to our study objective in the reviewed literature.

#### Sociodemographic questionnaire

We formulated questions regarding gender, age, type of settlement, highest educational level of parents, family structure, subjectively assessed financial status, number of siblings, exact description of chronic disease(s), and achievement of independent (without the parent) sleep.

#### *Connor–Davidson resilience scale*^29^

The questionnaire measures successful coping with stress, or the ability to recover from challenging circumstances. The person evaluates the statements related to of resilience on a 5-point Likert scale through a factor, scoring between 10 and 40 points (Cronbach’s α = 0.851).

#### *Satisfaction with life scale*^30^

This test consists of a 5-item scale that assesses one's overall cognitive evaluation of life satisfaction, rather than measuring positive or negative emotions. Participants express their level of agreement or disagreement with each item on a 7-point scale, ranging from strongly agree to strongly disagree (Cronbach’s α = 0.74).

#### *Cantril ladder*^31^

The Cantril Ladder serves as a visual tool for evaluating overall contentment and happiness in life. Individuals are shown an image of a ladder with numbers ranging from zero to ten. This scale represents the spectrum of life satisfaction, with zero representing the lowest level and ten representing the highest. Participants are then asked to consider their current life situation and position themselves on the ladder accordingly. If their score falls at or below four, it indicates a state of 'suffering', while a score of seven or higher signifies 'thriving'. Essentially, a higher score on the ladder corresponds to a greater sense of well-being and satisfaction with life (Cronbach’s α = 0.86).

#### *Non-productive thoughts questionnaire (NPT)*^32^

This is a single-factor scale for measuring ruminations and rumination in childhood, with scores ranging from 10 to 30. The scale uses 10 items to measure perseverative trait-like thoughts in the questionnaire. Participants were instructed to rate each item on a 3-point scale, with 1 indicating "not true," 2 indicating "sometimes true," and 3 indicating "often true." The scores on this scale reflected the frequency of nonproductive thoughts, with higher scores indicating a higher frequency (Cronbach's alpha = 0.84).

#### *Problematic internet use questionnaire (PIU-Q)*^33^

The questionnaire uses a five-point scale to assess the level of agreement with the provided statement. The obsession subscale delves into contemplation and fantasies revolving around the Internet, as well as the mental symptoms experienced when deprived of Internet access. The neglect subscale pertains to the disregard for daily tasks and fundamental necessities. Lastly, the control disorder subscale encompasses items that address challenges in regulating Internet usage (Cronbach's alpha = 0.84).

#### *Drawing version of pictorial representation of illness self-measure (PRISM-D)*^34,35^

Participants in the PRISM-D drawing test are provided with an A4-size sheet of paper containing a 7 cm yellow circle positioned in the lower right corner. This circle serves as a representation of the individual's self, mirroring the original PRISM test. Four subscales of the drawing test developed by the Hungarian working group of the PRISM test were considered: the distance between the yellow circle symbolising self and the circle symbolising illness, the average area of the circle representing illness (25.12 cm^2^), the number of circles representing youth resources, and the total area of the circles representing resources were compared.

#### *The Beck depression inventory—shortened scale (BDI—R, shortened version)*^36,37^

Depression severity was assessed using the BDI, a questionnaire consisting of 21 items. These questions capture emotions experienced over a recent period of time. Each statement is assigned a score from 0 to 3, resulting in a total score that can range from 0 to 84 (Cronbach's alpha = 0.82).

#### *The illness intrusiveness ratings scale (IIRS)*^38^

The IIRS is a concise tool that individuals can use to report their own experiences.

Originally designed to assist individuals dealing with chronic and potentially life-threatening illnesses, the IIRS can also be utilized by those with less severe health conditions. Consisting of 13 questions, this assessment prompts participants to evaluate the extent to which their illness and/or its treatment disrupt various aspects of their life that are crucial to overall quality of life.

To assess the level of intrusion caused by illness, participants utilize a seven-point scale that spans from 1 (minimal) to 7 (significant) (Cronbach's alpha = 0.86).

#### *The Spielberger state-trait anxiety questionnaire: child version (STAI-S-C and STAI-T-C, state-trait anxiety inventory for children, STAI-C)*^39,40^

The STAI test was developed based on the adult questionnaire of the same name to assess anxiety levels in school-age children. The STAI-T is a scale that evaluates an individual's overall emotional state, while the STAI-S measures their current anxiety level specifically related to a particular event. Both scales consist of 20 items, with response options ranging from 1 (not experiencing at all) to 4 (experiencing intensely) (Cronbach's alpha = 0.77).

#### *The strength and difficulty questionnaire (SDQ)*^41,42^

The questionnaire is designed to measure children's behavioural characteristics and abilities, and it shows a consistent picture compared to the parent version. It is a concise survey designed to evaluate behavioural patterns in individuals aged 2–17. The SDQ questionnaire inquires about a total of 25 characteristics, encompassing both negative and positive attributes. These characteristics are distributed across 5 subscales, each consisting of 5 items. The subscales measure emotional symptoms, conduct problems, hyperactivity, peer problems, and prosocial behaviour. The scores from the items in the first 4 subscales are combined to calculate a total difficulties score (Cronbach's alpha = 0.75).

### Statistical analysis

A cross-sectional study was performed with a matched control group. Descriptive statistics were generated from the questionnaire data. The descriptive statistics are graphical representations of the scale of each variable to characterise and visualise the distributions, both for the population as a whole and for each of the diseases studied. One-way analysis of variance (ANOVA) was used to examine differences between groups, and Pearson rank correlation analysis was used to measure the relationships between individual variables.

### Ethical approval and consent to participate

This research was conducted in accordance with the Declaration of Helsinki. The Institutional Research Ethics Committee—University of Debrecen Clinical Centre (REC—IKEB) approved this study (DE RKEB/IKEB 5858-2021). The research is conducted ethically, the results are reported honestly, the submitted work is original and not (self-) plagiarised, and authorship reflects the individuals' contributions. Informed consent was obtained from all subjects and/or their legal guardian(s).

## Results

### Sociodemographic characteristics of children

Overall, 107 children completed our questionnaire, with 58 boys (54.2%) and 49 girls (45.8%) in gender distribution. The mean age was 14.3 years (SD = 2.0). 48 (44.86%) live in county capitals, 12 (11.21%) in big cities, 28 (26.17%) in small towns and 19 (17.76%) in villages. This proportion can be considered ideal, as it was very important to us not only to get the opinion of the population living in the county town. Regarding birth order, one child has no siblings, 63 are first born, 26 are second born, and the remaining 17 are the third or later child in the family (Table 1).

83.8% of the respondents live with their parents, 9.1% with their mother, 3% with their father, 1% with a grandparent and 3% with a foster parent. 80.8% have a room of their own, and 19.2% do not have an own room at the moment. 78.8% sleep in their room, 11.1% share a room with their parents and 10.1% with their siblings. According to their subjective assessment of their financial situation, 84% say they live on a medium income, 13% on a low income, and 3% say they have a high income.

The distribution of patients was as follows: 34 (16.4%) from gastroenterology, 21 (10.1%) from onco-haematology, 19 (9.3%) from pulmonology and 33 (15.9%) from rehabilitation. 82 persons (76.64%) stated that there were no family members with a chronic disease among their immediate relatives. In comparison, 25 persons (23.36%) stated that there were children and/or adults with a chronic disease among their family members.

There is a significant difference in the distribution of the groups of ill and healthy children in terms of the mother's education (p = 0.001): mothers with tertiary education are over-represented in the group of healthy children, while mothers with a maximum of primary education are over-represented in the group of sick children. A similar trend is also observed for the educational attainment of fathers, although in this case, the difference in the distribution of the groups is not significant (p = 0.252).

### Psychological characteristics of children

**Figure 1:**
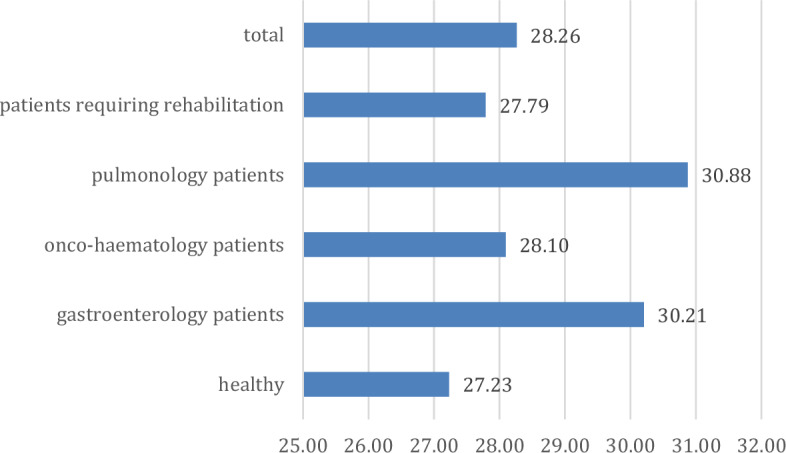
Distribution of resilience in the groups (points).

First, we tested H1 in which we supposed that children living with chronic illnesses report worse mental health status. The mean score for the whole sample regarding resilience was 28.26 (Fig. 1). Significant differences were found when comparing the groups (p = 0.040; η = 0.167; η^2^ = 0.028), with high scores for pulmonary and gastroenterological diseases. At the same time, below-average scores were also found for patients requiring rehabilitation, onco-haematological patients and healthy children. Patients requiring rehabilitation also show no difference in resilience levels compared to healthy children. They have coping resilience levels comparable to those of healthy children.

**Figure 2:**
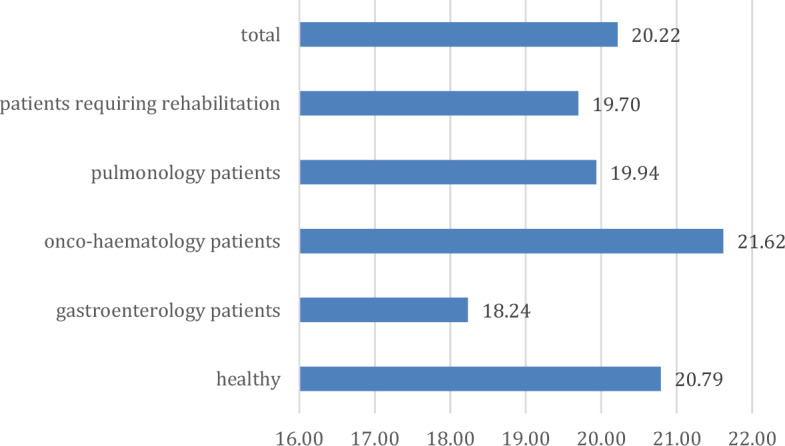
Non-productive thoughts scale scores across groups (points).

For Non-productive thoughts, the sample average is 20.22 points (Fig. 2). Compared to this mean, onco-haematological patients and healthy children showed higher scores. In contrast, children requiring rehabilitation diagnosed with pulmonary and gastrointestinal diseases showed lower scores, and although the difference is not significant, there is a trend level difference (p = 0.073; η = 0.189; η^2^ = 0.036).

For Problematic internet use, the overall sample scores for preoccupation with the Internet (obsession) were 3.28, neglect of daily activities (neglect) was 5.25, and difficulty controlling internet use (control) was 4.07. The scores are increasing, with neglect being the most significant phenomenon (Table 2). For obsession, there is a significant difference between groups (p = 0.001; η = 0.146; η^2^ = 0.021), with above-average scores for onco-haematology and pulmonology patients. In contrast, gastroenterology patients have the lowest and below-average scores for children requiring rehabilitation and healthy children. A trend level difference was found for the control dimension (p = 0.072; η = 0.143; η^2^ = 0.120) with the same pattern. For the dimension of neglect, the difference between groups was insignificant (p = 0.253; η = 0.193; η^2^ = 0.027).

**Table 2:** Dimensions of problematic Internet use across groups (points).

		Obsession**	Neglect	Control
Healthy	Mean	3.15	5.53	4.24
	Standard deviation	1.55	1.91	1.84
Gastroenterology Patients	Mean	2.82	5.15	3.47
	Standard deviation	1.14	1.58	0.99
Onco-Haematology Patients	Mean	4.29	5.14	4.24
	Standard deviation	2.24	2.10	2.17
Pulmonology Patients	Mean	4.06	5.31	4.81
	Standard deviation	2.11	2.75	2.74
Patients Requiring Rehabilitation	Mean	3.00	4.55	3.64
	Standard deviation	1.22	1.70	1.67
Total	Mean	3.28	5.25	4.07
	Standard deviation	1.66	1.95	1.86
P-Value		0.001	0.253	0.072
η		0.146	0.193	0.143
η^2^		0.021	0.027	0.120

The four main variables of the PRISM-D test were examined (Table 3). The average distance between the yellow circle representing self and the circle representing disease was 11.32 cm in the sample. In this case, there is a significant difference between groups. Significantly lower values were observed for onco-haematology and pulmonology, while significantly higher values were observed for gastroenterology (p = 0.036; η = 0.251; η^2^ = 0.063). The average number of circles representing the resources of young people was 3.34, with a significant difference between groups (p < 0.001; η = 0.469; η^2^ = 0.220), with a similar pattern to the previous one. The average area of the circle representing illness is 25.12 cm^2^, which is significant compared to the area of the circle representing self, 38.47 cm^2^. No significant differences are observed in this respect (p = 0.526; η = 0.158; η^2^ = 0.025). The overall area of the circles representing resources is 104.91 cm^2^. There is no significant difference for the circle representing self (p = 0.526; η = 0.212; η^2^ = 0.045), although above-average values are also observed for patients requiring rehabilitation, pulmonology and gastroenterology. For the area of the circles representing resources, there are also strikingly low values for onco-haematology, pulmonology and patients requiring rehabilitation, but the difference is not significant (p = 0.169).

**Table 3:** Dimensions of the PRISM-D test in the groups (points).

		PRISM SIS (cm)	PRISM IPM (cm)	PRISM KSZ (pcs)	PRISM KT (cm)^2^
Healthy	Mean	11.40	33.10	3.68	101,90
	Standard deviation	5.09	63.95	1.38	129,53
Gastroenterology Patients	Mean	16.22	34.85	4.09	183,99
	Standard deviation	23.82	125.87	0.80	337,43
Onco-Haematology Patients	Mean	7.16	10.63	2.33	42,69
	Standard deviation	7.87	19.72	2.18	62,42
Pulmonology Patients	Mean	13.50	24.22	3.88	82,12
	Standard deviation	6.83	28.75	1.80	69,06
Patients Requiring Rehabilitation	Mean	7.71	9.33	1.87	88,27
	Standard deviation	8.68	21.98	1.71	246,12
Full Sample	Mean	11.32	25.12	3.34	104,91
	Standard deviation	11.87	69.18	1.67	200,21
P-Value		0,036	0.526	< 0.001	0.169
η		0.251	0.158	0.469	0.212
η^2^		0.063	0.025	0.220	0.045

**Figure 3:**
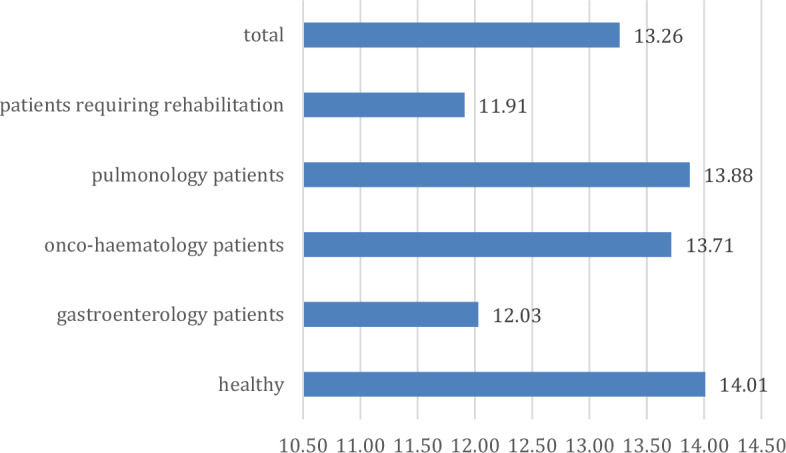
Trends in depression scores across groups (points).

For the Beck Depression Questionnaire, a mean of 4.54 points was found for the whole sample. The difference between the groups is at a trend level (p = 0.073; η = 0.221; η^2^ = 0.049), with the highest scores in pulmonology and haematology patients and healthy subjects and the lowest in patients requiring rehabilitation and gastroenterology patients (Fig. 3).

On the STAI scale of state anxiety to performance anxiety, a score of 45.51 was available, and the trait anxiety level was 32.29, according to children's self-report (Table 4). There was no significant difference between groups in scores on the STAI scale of situational anxiety—performance anxiety (p = 0.614; η = 0.132; η^2^ = 0.018), with scores above average for patients requiring rehabilitation and onco-hematologcal treatment, close to average for healthy children and below average for gastroenterology and pulmonology patients. The scores on the STAI general anxiety scale showed trend differences (p = 0.064; η = 0.221; η^2^ = 0.051), with the highest scores in healthy children and the lowest scores in patients with gastroenterology and those requiring rehabilitation.

**Table 4:** Dimensions of anxiety in the groups (points).

		STAI—state	STAI—trait
Healthy	Mean	45.55	34.11
	Standard deviation	9.965	8.295
Gastroenterology Patients	Mean	43.12	30.09
	Standard deviation	13.316	6.676
Onco-Haematology Patients	Mean	46.71	32.71
	Standard deviation	10.536	8.468
Pulmonology Patients	Mean	43.94	30.13
	Standard deviation	16.635	7.873
Patients Requiring Rehabilitation	Mean	47.76	30.55
	Standard deviation	8.359	7.710
Full Sample	Mean	45.51	32.39
	Standard deviation	11.011	8.081
P-Value		0,614	0.064
η		0.132	0.225
η^2^		0.018	0.051

The scores on the Strength and Difficulties Questionnaire scales were as follows: 4.55 points on the Hyperactivity Scale, 2.95 points on the Emotional Symptoms scale, 2.15 points on the Behavioural Problems scale, 4.67 points on the Peer Problems scale and 7.75 points on the Prosocial scale. Only the Emotional Symptoms scale showed a trend difference (p = 0.075; η = 0.220; η^2^ = 0.048), with higher scores for onco-hematology and pulmonology patients and lower scores for gastroenterology patients (Table 5).

**Table 5:** Dimensions of the Abilities and Difficulties Questionnaire in the groups (points).

		Hyperactivity scale	Emotional symptoms scale	Behavioural problems scale	Peer problems scale	Prosocial scale
Healthy	Mean	4.63	3.31	2.07	4.50	7.57
	Standard deviation	1.64	2.77	1.41	1.35	1.90
Gastroenterology Patients	Mean	4.68	2.12	2.03	4.59	7.65
	Standard deviation	1.57	2.06	1.09	1.16	1.52
Onco-Haematology Patients	Mean	4.48	3.57	2.33	4.86	7.71
	Standard deviation	2.20	2.99	1.83	1.53	1.98
Pulmonology Patients	Mean	4.71	3.25	2.38	5.13	8.63
	Standard deviation	2.28	3.36	1.41	1.50	1.67
Patients Requiring Rehabilitation	Mean	4.24	2.33	2.27	4.97	7.94
	Standard deviation	1.60	2.07	1.38	1.38	1.54
Full Sample	Mean	4.55	2.95	2.15	4.67	7.75
	Standard deviation	1.74	2.67	1.39	1.36	1.78
P-Value		0,674	0.075	0.898	0.356	0.273
η		0.125	0.220	0.090	0.027	0.176
η^2^		0.016	0.048	0.008	0.164	0.031

### The role of sociodemographic background variables in the development of mental health indicators

**Figure 4:**
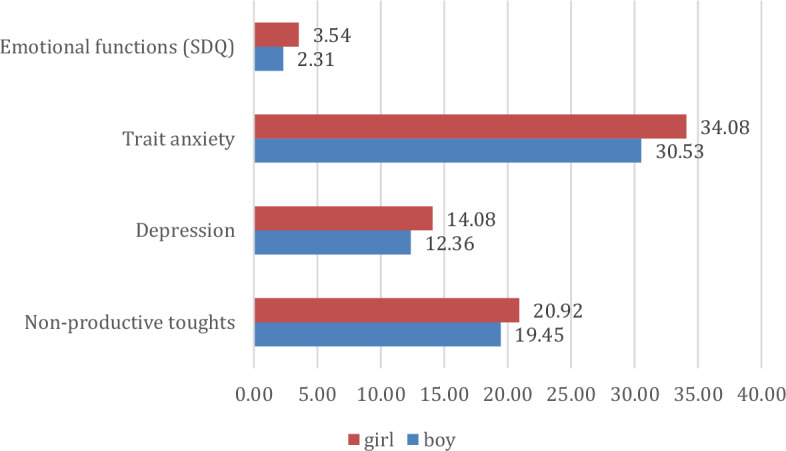
Major gender differences in mental health indicators (points).

Next, we tested H2, in which we supposed significant differences in the mental health status of children regarding the sociodemographic background variables. Thus, we examined which sociodemographic background variables play a significant role in the psychological variables. Significant and trend-level differences are presented along the trend of the results. Comparisons made to examine gender differences also highlighted some significant differences. For the Non-productive Thoughts Scale, higher scores for girls are seen (p = 0.025; η = 0.223; η^2^ = 0.049), as is the same trend for trait anxiety (p = 0.001; η = 0.312; η^2^ = 0.097), depression (p = 0.006; η = 0.218; η^2^ = 0.048) and emotional functioning (SDQ, p = 0.001; η = 0.196; η^2^ = 0.038). Further disease group-specific differences are not significant, presumably due to the rather small sample size (Fig. 4).

**Figure 5:**
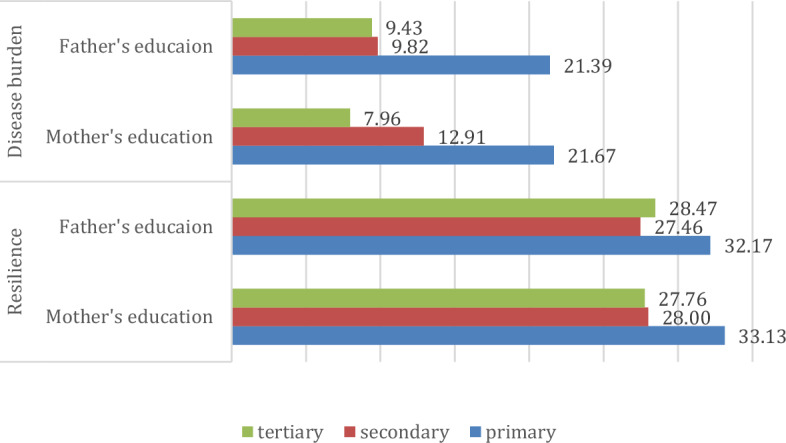
Role of parental education on mental health indicators (points).

We then examined the role of parental education on each mental health indicator (Fig. 5). Overall, a significant difference was found for resilience (maternal education: p = 0.006; η = 0.286; η^2^ = 0.082 paternal education: p = 0.011; η = 0.235; η^2^ = 0.055), which indicates that children of parents with low education have higher resilience scores. In addition, significant differences were found for the disease burden (mother’s education: p < 0.001; η η = 0.264; η^2^ = 0.070; father’s education: p = 0.001; η = 0.291; η^2^ = 0.085), which is also highest among children of parents with low education. Further disease group-specific differences are not significant, presumably due to the rather small sample size. Thus, children of parents with lower educational attainment had a higher burden of disease and were also the most reactive.

**Figure 6:**
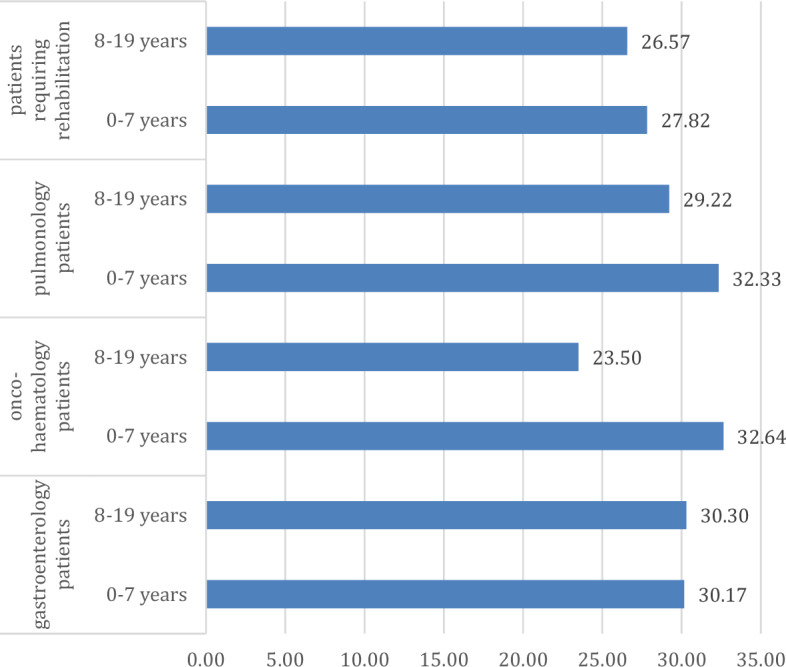
Evolution of resilience values across patient groups according to age (points).

**Figure 7:**
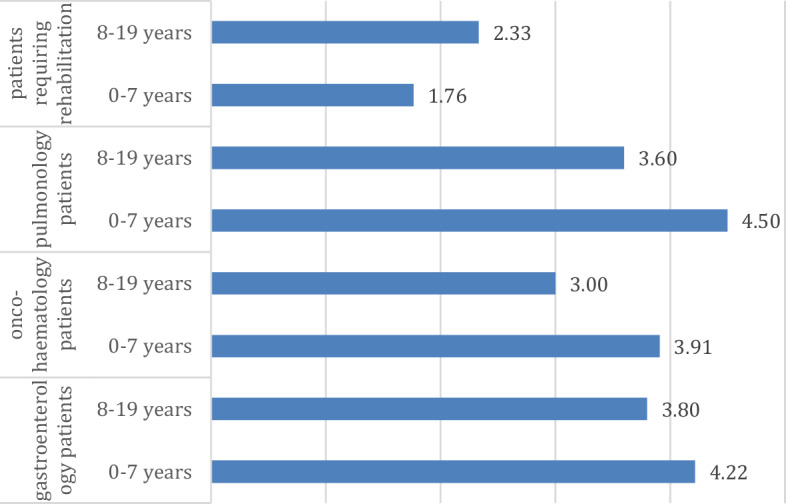
Trends in PRISM-D test round counts by age in patient groups (points).

Finally, we looked at the differences that could be assumed based on age at diagnosis, for which we created two groups: a group of patients diagnosed before the age of 7 and a group of patients diagnosed after the age of 7. The cases of relapses and projective drawing tests should be highlighted. In the case of resilience (p = 0.084; η = 0.132; η^2^ = 0.017), it can be seen that a diagnosis at a younger age can have a positive effect on the development of resilience, as higher resilience values were consistently found in young people diagnosed at an age younger than seven years (Fig. 6). For the PRISM-D test, a significant difference is seen in the number of circles (p < 0.001; η = 0.188; η^2^ = 0.035), except for gastroenterology patients (where almost the same numbers are seen in both groups), where more circles drawn for children with a diagnosis at a younger age indicates that there are more helping relationships and more resilience for children who "get into" their illness from a younger age (Fig. 7).

### Interactions between the main variables

To explore the relationships more deeply and test H3, we have also sought to explore the relationships between our main variables, of which the significant correlations are described. The correlation coefficients between satisfaction with life (SWLS) and the Cantril Ladder current and projected improvement in condition five years later are r = 0.478 (p < 0.001) and r = 0.476 (p < 0.001), respectively, indicating a remarkably significant coefficient and positive correlation between the two tests. Both more positive current state of life satisfaction and belief and confidence in the future may be related according to the results.

The Satisfaction with Life Scale (SWLS) also shows a positive correlation with the Connor Davidson Resilience Scale (r = 0.487, p < 0.001), suggesting that an important coefficient in children's lives is the development of appropriate resilient behaviour and that there may be an inverse correlation between them. Children with more resilience are also more satisfied with their quality of life.

The Connor Davidson Resilience Scale shows a negative correlation with Non-productive Thoughts (r = − 0.285, p < 0.001), which is an evidential correlation. Depressive mood has a weak positive relationship with the indicators of basic well-being (r = 0.312, p = 0.001). However, a weaker negative relationship is observed with general daily well-being (r = − 0.276, p = 0.004). Within the test, the five indicators of basic well-being and the **combined perception are also correlated (r = − 0.487, p < 0.001).

For age, we found a not very strong but significant relationship in three places. The first scale of the Cantril ladder, where children were asked to interpret their current life situation on a scale of 0–10, yielded a slight negative correlation (r = − 0.198, p < 0.042). Even age showed a stronger association with the scales of the SDQ questionnaire, with two out of four scales of the Abilities and Difficulties Questionnaire (the Behavioural Problems scale: r = − 0.298, p < 0.001, and the Prosocial scale: r = − 0.169, p = 0.015) showing an inverse association. These suggest that difficulties in the emotional and social domains and their perception may increase with age and may be exacerbated by age-specific characteristics.

## Discussion

The demographic data show that almost half of the respondents live in the county capital, while the other half live in smaller settlements scattered throughout the county, thus reflecting local conditions. The proportion of first-borns among children with chronic illnesses is very high. As the proportion of children with siblings is almost 100%, this can place an even greater burden on families and siblings alike^43^.

Regarding H1, where we supposed that healthy children possess better mental health status compared to chronically ill children, an ambivalent picture was uncovered. In terms of the psychological characteristics of each patient group, there is no consistent trend towards which disease group is most at risk of certain types of mental disorders, as in most of the psychological correlates examined, no significant difference was found between disease groups or between healthy controls. However, an interesting trend is that for some variables, the healthy groups scored below the desired values and, contrary to expectations, scored worse than the patient groups which means that H1 had to be rejected. This may be due to the fact that the different patient groups may receive a higher level of possibly targeted psychological counselling and support and may also receive a higher level of support from parents and peers^44^. A further explanatory factor may be their sociodemographic situation, as the healthy patient group may come from a fundamentally better social environment with higher levels of parental education, which may be associated with expectations of higher academic and non-academic achievement factors (e.g. more academic achievement, expectations of behaviour in line with social norms), which may result in a deterioration in mental health indicators^25–28^.

There is also considerable variation in the types of disease. Children receiving rehabilitation care show fewer intellectual symptoms, cope better with difficulties, and have a less volatile emotional system. The results, therefore, suggest that children in rehabilitation care are emotionally sensitive but slower to respond, which is an excellent symbol for the psychological manifestation of their physical symptom complex. It should be pointed out that patients requiring rehabilitation do not differ from healthy individuals in their level of resilience. They, therefore, have at least the resilience of healthy children. Resilience, as a psychosocial skill that reduces negative emotions while promoting adjustment during a crisis^45^, plays an important role in maintaining and improving patients' mental health so that they can engage in behaviours that help them cope with the anxiety and depression caused by their life-threatening chronic illness and ultimately improve their quality of life^46^.

The distributions for non-productive thoughts follow the evolution of the resilience level well. The higher score for healthy children also indicates a lower presence of psychological support^47^. In terms of self-reporting, self-blame and recurrent ruminative thoughts such as "Why did this happen to me? How did I get this disease?" may be more typical for children. The same may be the case for children with onco-haematological disease, which may be due to the severity of the disease. Previous research has already highlighted the presence of a negative health thought spiral in children diagnosed with severe illness^8–11,48,49^. The higher dependence on the Internet among onco-haematology patients also supports the former findings. The Internet can even be used in the therapeutic process, for example, in cancer patients, as successfully applied in the study by Albert et al.^50^, but the key is the quantity. The literature highlights that some types of disease, e.g. eating disorders, may be characterised by higher levels of dependence on the Internet^51^. This may be due to societal expectations mediated by the media and maladaptive coping strategies.

For the PRISM-D test, the distance between self and disease varied by disease type, with gastroenterological diseases being the most significant. A study by Munkácsi et al.^52^ among children diagnosed with type 1 diabetes highlighted the role of time since diagnosis and early diagnosis, which was not confirmed in the present study for gastroenterology or other disease types. In addition to these findings, there was a significant difference between groups in the number of circles presenting resources for young people, with a similar pattern to the previous one, i.e. a higher level of support was found among gastroenterological patients. In contrast, the lowest was found among children requiring rehabilitation. However, in their case, due to the more positive resilience, anxiety and depression scores, it can be assumed that they feel less need for stress from external resources.

There is also a trend in the distribution of depression scores, with a lower prevalence of depression among young people with gastroenterological and rehabilitation-related conditions. This may be due to the severity of the disease, its management options and the quality of life and vision available, which may be more positive for these diseases than other diseases, such as onco-haematological diseases. In cases where there are multiple co-morbidities, there are higher levels of depression compared to patients diagnosed with a single disease, particularly if it is a physical (motor) disease^15,16,53^.

In terms of anxiety, situation-specific anxiety does not show any relevant differences between disease groups or between healthy and ill children. In contrast, the overall level of anxiety was no longer consistent, and differences were not hypothesised for each disease type but between healthy and chronically ill children. This confirms our findings on resilience, highlighting the need for psychological interventions and health promotion with healthy children^54^. In terms of coping (skills and difficulties), we find trend differences in the management of emotional symptoms, which is prominent in children with onco-hematological and pulmonological diseases, with higher emotion-based coping in these disease types paralleling their higher depression and lower resilience scores, which is symbolic of the nature of their disease^55^.

Regarding H2, we can state that research findings on sociodemographic background are in line with previous findings. Regarding gender, we could see some important differences since the level of non-productive thoughts, trait anxiety, depression^56^ and emotional functioning was higher among girls. Several research highlighted the worse mental health status of girls, especially in adolescence. Girls typically can be characterised with lower level of self-esteem and self-confidence, higher level of anxiety, stress, ruminative and non-productive thoughts. The use of emotion-focused coping strategies is also more frequent among them^57,58^.

The distribution of resilience scores for parental educational attainment illustrates the nature of resilience as a psychological variable with disadvantage-compensating characteristics. In this light, the evolution of disease burden scores among children of parents with low educational attainment may be surprising, indicating that coping with disease is a major challenge for young people, who are, however, able to build coping skills (also building on other life domains) to cope with difficulties related to disease and other domains in a more resilient way^27,28,58–60^.

Previous findings also suggest that the age of the diagnosis is increasingly important. It allows for timely intervention and management, preventing the progression of the condition to more severe stages. Early detection also facilitates better treatment outcomes, improving the quality of life for individuals. Moreover, it enables individuals to make informed lifestyle choices and adopt preventive measures, reducing the overall impact of the illness^14,61^. Our research also confirmed that having an early diagnosis (depending on the illness itself) can have a positive effect on the development of resilience, as young people diagnosed at an age younger than seven years reported higher resilience. Overall, our H2 was partly confirmed.

Last but not at least, H3 explored the relationship between the different mental health-related variables. Satisfaction with life and perceptions of current life situation are strongly correlated, closely and positively related to a confident attitude towards the future. Those who are more optimistic about their life situation in the present, including their chronic illness, are also more positive about their future outlook^62^. The relationship between depressed mood and general well-being seems to suggest that subjects significantly distort their characterisation of their current state when presented in detail and when presented as a whole. A bias is observed because they show improved health in a more positive direction.

For age, there was a slight negative association with the current living situation, which differs from the previous trend (e.g.^63^). Younger children may rate their current living situation with the burden of their illness as less favourable than their older counterparts. No firm conclusions can be drawn on the strength of the relationship, but in any case, age no longer influences the perception of the future five years later when asked about the life situation. This may be due to their cognitive development, the quality and quantity of their social relationships, and the presence and depth of adolescent crises. The correlation between age and the Strength and Difficulties Questionnaire suggests that difficulties in the emotional and social domains increase with age and that age-related characteristics may exacerbate their perception^64^. Therefore, H3 was also partly confirmed.

### Strengths and limitations

One of the strengths of the research is that it explores a topic that, despite its high relevance, is still a relatively neglected research field. The research findings can be implemented well in primary and specialised care practice (e.g. changing the framework for psychological counselling in hospital wards) but also provide relevant experience for system development at the top (health policy) level.

The limitations of our research are also partly based on voluntary participation and reduced motivation, as the topic may arouse resistance among subjects. For this reason, it should be mentioned that the research is not representative. It is worth considering in further research the possibility of completing the questionnaires online (online completion may be problematic due to the high error factor, which can be effectively reduced by face-to-face presence). Furthermore, the PRISM-D projective test, which is included in the questionnaire, is a complex IT task to complete and fill in online. There is also a limitation in using paper–pencil tests; self-reporting is often biased when forming an opinion of oneself. Often, especially in adolescence, young people focus not only on the truth but also on what is the most correct and expected answer from them.

## Conclusions

Previous studies focusing on chronic diseases have already highlighted the importance of early diagnosis concerning mental health, such as quality of life. In parallel, the present study shows a positive manifestation of early diagnosis regarding resilience and social networks. Furthermore, the results highlight that close collaboration of a multidisciplinary team can have a positive impact on the mental health of children and young people, and we, therefore, propose to support and implement the national good practice of these teams, which would allow a sustained collaboration of GPs, psychologists, physiotherapists, dieticians, etc. In the Hungarian context, it should also be pointed out that the names of the institutions and social attitudes still convey a negative image, so parents are reluctant to send their children to specialist psychological care.

## Data Availability

Data are available only on request due to ethical restrictions. For further information, please contact the following email address: kovacs.karolina@arts.unideb.hu.

## Abbreviations

BDI: Beck depression inventory
CDR: Connor–Davidson resilience questionnaire
IIRS: Illness intrusiveness ratings scale
NPT: Non-productive thoughts questionnaire
PIU: Problematic internet use questionnaire
PRISM-D: Drawing version of pictorial representation of illness self-measure
SDQ: Strength and difficulty questionnaire
STAI: Spielberger state-trait anxiety
SWLS: Satisfaction with life scale

## References

[1] Kujala UM, Hautasaari P, Vähä-Ypyä H (2019). Chronic diseases and objectively monitored physical activity profile among aged individuals—A cross-sectional twin cohort study. Ann. Med..

[2] Viswanathan M, Golin CE, Jones CD (2012). Interventions to improve adherence to self-administered medications for chronic diseases in the united states: A systematic review. Ann. Intern Med..

[3] Brown MT, Bussell JK (2011). Medication adherence: Who cares?. Mayo Clin. Proc..

[4] World Health Organization (2003). Adherence to Long-Term Therapies: Evidence for Action.

[5] Perocier VL (2023). Severe mental illness and family involvement during treatment. Fam. J..

[6] Modi AC, Pai AL, Hommel KA (2012). Pediatric self-management: A framework for research, practice, and policy. Pediatrics..

[7] Stein REK (2022). Mental health concerns and childhood chronic physical health conditions: A narrative review. Pediatr Med..

[8] Lebel S, Mutsaers B, Tomei C (2020). Health anxiety and illness-related fears across diverse chronic illnesses: A systematic review on conceptualization, measurement, prevalence, course, and correlates. PLoS One..

[9] Nakao M, Shirotsuki K, Sugaya N (2021). Cognitive-behavioral therapy for management of mental health and stress-related disorders: Recent advances in techniques and technologies. Biopsychosoc. Med..

[10] Correale C, Falamesca C, Tondo I (2022). Depressive anxiety symptoms in hospitalized children with chronic illness during the First Italian COVID-19 lockdown. Children (Basel)..

[11] Barker MM, Beresford B, Fraser LK (2023). Incidence of anxiety and depression in children and young people with life-limiting conditions. Pediatr Res..

[12] Spurr S, Danford CA, Roberts KJ (2023). Fathers' experiences of caring for a child with a chronic illness: A systematic review. Children (Basel)..

[13] Baghdadi LR, Alsaiady MM (2024). Medication adherence barriers and their relationship to health determinants for saudi pediatric dialysis patients. Children.

[14] Colizzi M, Lasalvia A, Ruggeri M (2020). Prevention and early intervention in youth mental health: Is it time for a multidisciplinary and trans-diagnostic model for care?. Int. J. Ment. Health Syst..

[15] Montag C, Demetrovics Z, Elhai JD (2024). Problematic social media use in childhood and adolescence. Addict. Behav..

[16] El-Ashry AM, Hussein Ramadan Atta M, Alsenany SA, Farghaly Abdelaliem SM, Abdelwahab Khedr M (2023). The effect of distress tolerance training on problematic internet use and psychological wellbeing among faculty nursing students: A randomized control trial. Psychol Res Behav Manag..

[17] Mateo-Orcajada A, Vaquero-Cristóbal R, Albaladejo-Saura MD, Abenza-Cano L (2023). The degree of problematic technology use negatively affects physical activity level, adherence to mediterranean diet and psychological state of adolescents. Healthcare..

[18] van der Laan SEI, van der Berkelbach Sprenkel EE, Lenters VC, Finkenauer C, van der Ent CK, Nijhof SL (2023). Defining and measuring resilience in children with a chronic disease: A scoping review. Advers. Resil. Sci..

[19] Southwick SM, Bonanno GA, Masten AS, Panter-Brick C, Yehuda R (2014). Resilience definitions, theory, and challenges: Interdisciplinary perspectives. Eur. J. Psychotraumatol..

[20] Kunzler AM, Helmreich I, Chmitorz A (2020). Psychological interventions to foster resilience in healthcare professionals. Cochr. Database Syst. Rev..

[21] Xu J, Zhang L, Sun H (2023). Psychological resilience and quality of life among middle-aged and older adults hospitalized with chronic diseases: Multiple mediating effects through sleep quality and depression. BMC Geriatr..

[22] Gautam N, Dessie G, Rahman MM, Khanam R (2023). Socioeconomic status and health behavior in children and adolescents: A systematic literature review. Front. Public Health..

[23] Reiss F, Meyrose AK, Otto C, Lampert T, Klasen F, Ravens-Sieberer U (2019). Socioeconomic status, stressful life situations and mental health problems in children and adolescents: Results of the German BELLA cohort-study. PLoS One..

[24] Ahad AA, Sanchez-Gonzalez M, Junquera P (2023). Understanding and addressing mental health stigma across cultures for improving psychiatric care: A narrative review. Cureus..

[25] Behere AP, Basnet P, Campbell P (2017). Effects of family structure on mental health of children: A preliminary study. Indian J Psychol Med..

[26] Chen X, Orom H, Hay JL (2019). Differences in rural and urban health information access and use. J. Rural Health..

[27] Scrimin S, Mastromatteo LY, Hovnanyan A, Zagni B, Rubaltelli E, Pozzoli T (2022). Effects of socioeconomic status, parental stress, and family support on children's physical and emotional health during the COVID-19 pandemic. J. Child Fam. Stud..

[28] Kraft P, Kraft B (2021). Explaining socioeconomic disparities in health behaviours: A review of biopsychological pathways involving stress and inflammation. Neurosci. Biobehav. Rev..

[29] Járai, R., Vajda, D., Hargitai, R., László, N., Csókási, K., & Kiss, E.C. A Connor–Davidson Reziliencia Kérdőív 10 itemes változatának jellemzői [Features of the 10-item version of the Connor–Davidson Resilience Questionnaire]. *Alkalmazott Pszichológia*. **15**(1), 129–136 (2015). 10.17627/ALKPSZICH.2015.1.129

[30] Martos T, Sallay V, Désfalvi J, Szabó T, Ittzés A (2014). Psychometric characteristics of the hungarian version of the satisfaction with life scale (Swls-h). Mentálhigiéné és Pszichoszomatika..

[31] Cantril H (1965). The Pattern of Human Concerns.

[32] Kocsel N, Mónok K, Szabó E (2019). Gender invariance and psychometric properties of the nonproductive thoughts questionnaire for children. Assessment.

[33] Demetrovics, Z. S., Szeredi, B., & Nyikos, E. Presentation of the questionnaire on problematic internet Use. *Psyichiatria Hungarica*. **19**(2), 141–160 (2014).

[34] Büchi S, Sensky T (1999). Prism: Pictorial representation of illness and self measure. Psychosomatics.

[35] Havancsák, R., Pócza-Véger, P., Csabai, M. The PRISM-D drawing test in the examination and treatment of hospital patients [A PRISM-D rajzteszt kórházi betegek vizsgálatában és kezelésében]. In: Csabai M, Pintér JN, editors. Psychology in healing: Phenomenological, art psychological and body representation centered approaches [Pszichológia a gyógyításban: Fenomenológiai, művészetpszichológiai és test-kép-központú megközelítések]; 83–107 (2013).

[36] Beck AT, Beck RW (1972). Screening depressed patients in family practice: a rapid technic. Postgrad. Med..

[37] Rózsa S, Szádóczky E, Füredi J (2001). A Beck Depresszió Kérdőív rövidített változatának jellemzői a hazai mintán [Characteristics of the shortened version of the Beck Depression Questionnaire in a national sample]. Psychiatria Hungarica..

[38] Devins GM, Edworthy SM, Seland TP, Klein GM, Paul LC, Mandin H (1993). Differences in illness intrusiveness across rheumatoid arthritis, end-stage renal disease, and multiple sclerosis. J. Nerv. Ment. Dis..

[39] Spielberger CD (1973). Manual for the state trait anxiety inventory for children.

[40] Sipos, K., & Sipos, M. Standardization and validation of the "State-Trait Anxiety Inventory for Children". In *Hungarian. Theoretical-Methodological Studies* (Institute of Psychology, Hungarian Academy of Sciences, Budapest, 1979).

[41] Goodman R (1997). The strengths and difficulties questionnaire: a research note. Child Psychol. Psychiatry..

[42] Birkás E, Lakatos K, Tóth I (2008). Gervai J Gyermekkori viselkedési problémák felismerésének lehetőségei rövid kérdőívekkel: A Strengths and Difficulties Questionnaire Magyar változata [Identifying childhood behavioural problems with short questionnaires: The Hungarian version of the Strengths and Difficulties Questionnaire]. Psychiatria Hungarica..

[43] Pereira L, Radovic T, Haykal KA (2021). Peer support programs in the fields of medicine and nursing: A systematic search and narrative review. Can Med Educ J..

[44] Kiss, C. S. Malignant hematological and oncological diseases. In Tulassay T., editor. *Clinical Pediatrics*. (Medicina Könyvkiadó Zrt., Budapest, 2018).

[45] Rohan JM, Verma T (2020). Psychological considerations in pediatric chronic illness: Case examples. Int. J. Environ. Res. Public Health..

[46] Yoo KH (2006). A correlational study on the mastery and depression in chronic arthritis patients. Kor. J. Rehabil. Nurs..

[47] Sandler IN, Wolchik SA, Ayers TS (2007). Resilience rather than recovery: A contextual framework on adaptation following bereavement. Death Studies..

[48] Lopez-Rodriguez MM, Fernández-Millan A, Ruiz-Fernández MD, Dobarrio-Sanz I, Fernández-Medina IM (2020). New technologies to improve pain, anxiety and depression in children and adolescents with cancer: a systematic review. IJERPH..

[49] Scott K, Lewis CC, Marti CN (2019). Trajectories of symptom change in the treatment for adolescents with depression study. J. Am. Acad. Child Adolesc. Psychiatry..

[50] Alberts NM, Hadjistavropoulos HD, Titov N, Dear BF (2018). Patient and provider perceptions of Internet-delivered cognitive behavior therapy for recent cancer survivors. Supp. Care Cancer..

[51] Wang J, Hao QH, Peng W, Tu Y, Zhang L, Zhu TM (2023). Relationship between smartphone addiction and eating disorders and lifestyle among Chinese college students. Front. Public Health..

[52] Munkácsi B, Papp G, Felszeghy E, Kovács KE, Nagy BE (2018). The associations between mental health, health-related quality of life and insulin pump therapy among children and adolescents with type 1 diabetes. J. Pediat. Endocrinol. Metab..

[53] Read JR, Sharpe L, Modini M, Dear BF (2017). Multimorbidity and depression: A systematic review and meta-analysis. J. Affect. Disord..

[54] Koné Pefoyo AJ, Bronskill SE, Gruneir A (2015). The increasing burden and complexity of multimorbidity. BMC Public Health..

[55] Hetrick, S. E., McKenzie, J. E., Bailey, A. P., *et al*. New generation antidepressants for depression in children and adolescents: A network meta-analysis. *Cochrane Common Mental Disorders Group, ed. Cochrane Database of Systematic Reviews*. 2021(5) (2021). doi:10.1002/14651858.CD013674.pub2 10.1002/14651858.CD013674.pub2 PMC8143444 34029378

[56] Graves BS, Hall ME, Dias-Karch C, Haischer MH, Apter C (2021). Gender differences in perceived stress and coping among college students. PLoS ONE.

[57] Johnson DP, Whisman MA (2013). Gender differences in rumination: A meta-analysis. Pers. Individ. Dif..

[58] Ceglédi T, Fényes H, Pusztai G (2022). The effect of resilience and gender on the persistence of higher education students. Soc. Sci..

[59] Alduraidi H, Dardas LA, Price MM (2020). Social determinants of resilience among syrian refugees in jordan. J. Psychosoc. Nurs. Ment. Health Serv..

[60] Schickedanz A, Halfon N (2020). Evolving roles for health care in supporting healthy child development. Future Child..

[61] Avvenuti G, Baiardini I, Giardini A (2016). Optimism’s explicative role for chronic diseases. Front. Psychol..

[62] Sirois FM (2015). Who looks forward to better health? Personality factors and future self-rated health in the context of chronic illness. Int. J. Behav. Med..

[63] Maslow GR, Haydon AA, Ford CA, Halpern CT (2011). Young adult outcomes of children growing up with chronic illness: An analysis of the National Longitudinal Study of Adolescent Health. Arch. Pediatr. Adolesc. Med..

[64] Iovino P, Vellone E, Cedrone N, Riegel B (2023). A middle-range theory of social isolation in chronic illness. Int. J. Environ. Res. Public Health..

